# Case Report: One-Year Delay in the Effect of Conversion Surgery Therapy for Advanced Hepatocellular Carcinoma After Systemic Therapy

**DOI:** 10.3389/fmolb.2021.810251

**Published:** 2022-02-04

**Authors:** Qing-Yu Xie, Hai-Yan Liu, Ze-Yi Guo, Yan-Ping Wu, Guo-Lin He, Lei Cai, Ming-Xin Pan, Shun-Jun Fu

**Affiliations:** Department of Hepatobiliary Surgery II, General Surgery Center, Guangdong Provincial Research Center for Artificial Organ and Tissue Engineering, Guangzhou Clinical Research and Transformation Center for Artificial Liver, Institute of Regenerative Medicine, Zhujiang Hospital, Southern Medical University, Guangzhou, China

**Keywords:** hepatocellular carcinoma, conversion therapy, camrelizumab, portal vein thrombosis (PVTT), operation

## Abstract

Hepatocellular carcinoma (HCC) is the sixth most commonly diagnosed malignancy and the third leading cause of cancer-related deaths worldwide. A 58-year-old man visited his local hospital due to abdominal discomfort and was diagnosed with lung metastasis. After admission to our hospital in April 2020, he received two cycles of transcatheter arterial embolization (TAE), hepatic arterial infusion chemotherapy (HAIC-Folfox), sorafenib, and camrelizumab every 3 weeks. Due to the end of HAIC treatment, he underwent drug-eluting transcatheter arterial chemoembolization (dTACE) once, sorafenib, and camrelizumab. However, because of worsening liver function, we interrupted TACE and only gave sorafenib and camrelizumab in August 2020. Although he received systemic therapy, the tumors still rapidly progressed and we considered the possibility of tumor resistance. Subsequently, regorafenib was given. In September, the patient underwent conventional TACE (cTACE) once, regorafenib, and camrelizumab. After half a year of comprehensive treatment, the treatment effect was not satisfactory, and he returned to the local hospital to received regorafenib every day and camrelizumab once every 3 weeks. The patient found that the tumor and lung metastasis had shrunk significantly after 1 year of the initial diagnosis, then he was admitted to our hospital and received surgery treatment, and now he has survived disease-free for 6 months.

## Introduction

Hepatocellular carcinoma (HCC) is the sixth most commonly diagnosed malignancy and the third leading cause of cancer-related deaths worldwide (Sung et al., 2021). In China, HCC is the fourth most commonly diagnosed and the second leading cause of cancer-related deaths (Chen et al., 2016). As we all know, the best treatment for HCC is radical surgical resection. Unfortunately, most patients with HCC are diagnosed at an advanced stage. In recent years, a new treatment called “conversion surgery therapy” has been reported that converts unresectable gastrointestinal cancer to resectable gastrointestinal cancer, which has improved the prognosis of patients (Sato et al., 2017).

We herein report a case of advanced HCC that received conversion therapy up to a year before it took effect, and finally performed a successful operation.

## Materials and Methods

We retrospectively reported and analyzed a case that pathologically confirmed primary HCC at Zhujiang Hospital. The clinical data including clinical symptoms, signs, pathological diagnosis, radiological findings, laboratory analyses, treatments, and outcome were obtained from the hospital’s electronic medical records. Pathological diagnosis including immunohistochemistry (IHC) was independently reviewed by two pathologists. The patient’s family approved the anonymous use of the patient’s data, which was in accordance with the Helsinki Declaration.

## Results

### Case Report

A 58-year-old male patient complained abdominal discomfort for 2 months. He went to his local hospital and was diagnosed with HCC and Barcelona Clinic Liver Cancer (BCLC) stage C in March 2020. Then, transcatheter arterial chemoembolization (TACE) was performed.

**FIGURE 1:**
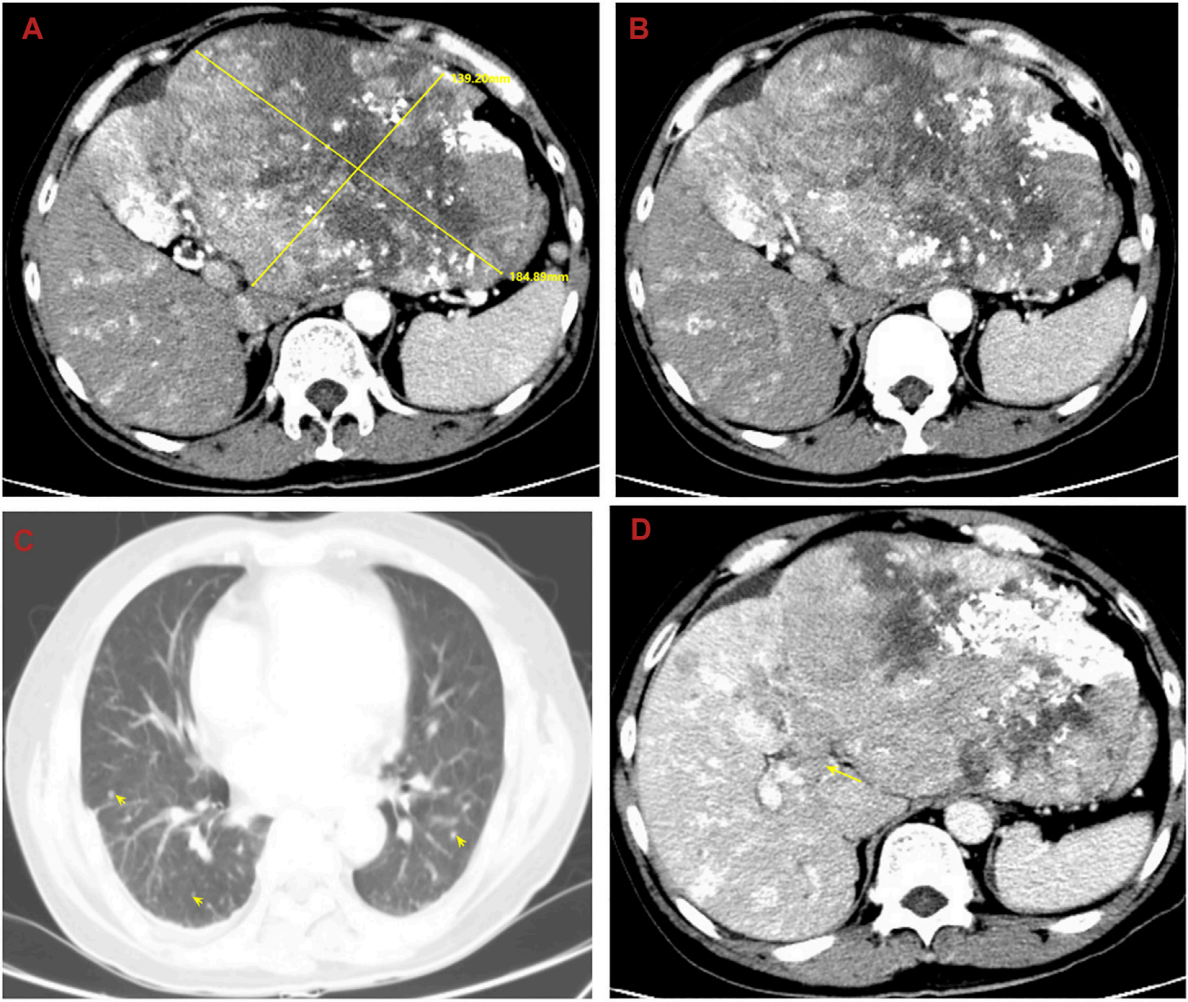
Abdominal computed tomography (CT) shows the tumor in the left lobe of the liver considered to be HCC with multiple metastases in the right lobe of the liver, and with tumor thrombosis in the left portal vein and lung metastases. **(A)** The huge tumor in the left lobe of the liver; **(B)** the multiple metastases in the right lobe of the liver; **(C)** the lung metastases; **(D)** the tumor thrombosis in the left portal vein.

**FIGURE 2:**
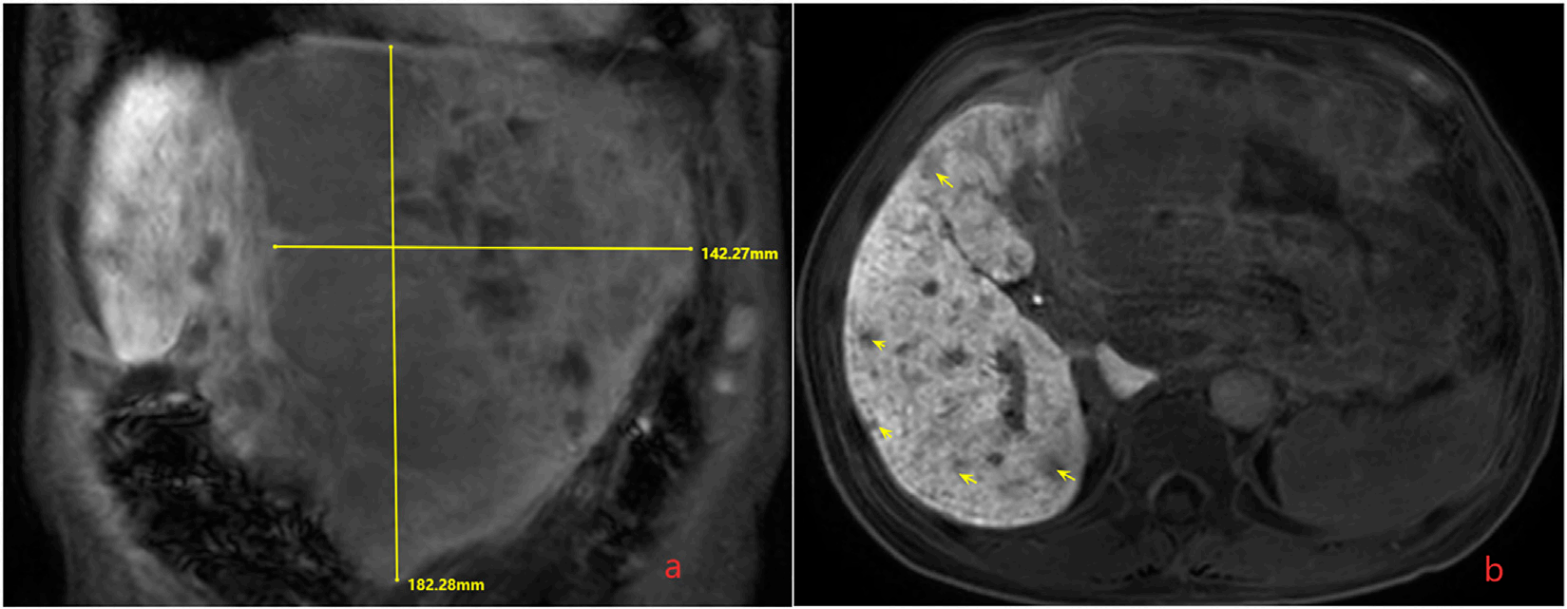
Magnetic resonance imaging (MRI) shows the tumor in the left lobe of the liver with multiple metastases in the liver, and with tumor thrombosis in the left portal vein. **(A)** The huge tumor in the left lobe of the liver; **(B)** the multiple metastases in the right lobe of the liver.

In order to seek further diagnosis and treatment, he went to our hospital and was admitted in April 2020. He had a history of hepatitis B. On admission, physical examination showed tenderness over the right upper region area with a 5 cm palpable abdominal mass below the xiphoid process. Laboratory data were recorded as follows: alanine aminotransferase (ALT): 36 IU/L, aspartate transaminase (AST): 81 IU/L, albumin (ALB): 22.7 g/L, total bilirubin (TBIL): 9.7 μmol/L, prothrombin time (PT): 11.5 s, and HBV-DNA: 6.83 × 10^2^ IU/ml. Among the tumor markers, alpha fetoprotein (AFP) was significantly elevated (1,649.0 μg/L), carbohydrate antigen 125 (CA125) was sightly increased (113.0 kU/L), and carcinoembryonic antigen (CEA) and carbohydrate antigen 199 (CA199) were normal. The cirrhosis was classified as Child-Pugh 7 and ALBI grade 3. The enhanced CT revealed small nodules in the middle and lower lobes of the lungs that were considered lung metastases, the tumor in the left lobe of the liver was considered HCC with multiple metastases in the right lobe of the liver, and tumor thrombosis was present in the left portal vein (Figure 1). The magnetic resonance imaging (MRI) showed the tumor in the left lobe of the liver with multiple metastases in the right lobe of the liver, and with tumor thrombosis in the left portal vein (Figure 2).

1. After MDT discussion, HCC with BCLC stage C and portal vein tumor thrombus (PVTT) classified VP3 and Cheng’s type II was diagnosed, and we believe that radical surgical resection was impossible due to the low residual liver volume and lung metastases. He finally received conversion therapy. The procedure details are as follows: The patient received two cycles of transcatheter arterial embolization (TAE), hepatic arterial infusion chemotherapy (HAIC-Folfox) (the HAIC scheme was as follows: We selectively placed a microcatheter into the feeding arteries of the tumor. Then, the microcatheter was connected to the artery infusion pump to perform the following treatment: Day 1: oxaliplatin 85 mg/m^2^, leucovorin 400 mg/m^2^, and 5-fluorouracil 400 mg/m^2^ via intra-arterial infusion; day 2–3: 5-fluorouracil 2,400 mg/m^2^ via continuous intra-arterial infusion. After HAIC was completed, the catheter was removed, and compression was performed to achieve hemostasis. The patient was treated every 3 weeks) (He et al., 2019), sorafenib (at a dose of 400 mg twice daily), and camrelizumab (200 mg intravenously every 3 weeks). At the end of HAIC treatment, we were disappointed to find that the tumor had rapidly progressed based on the modified Response Evaluation Criteria in Solid Tumors (mRECIST). Then, the patient received drug-eluting TACE (dTACE) once, sorafenib, and camrelizumab in July 2020. A month later, his liver function deteriorated (ALT: 33 IU/L, AST: 329 IU/L, ALB: 24.9 g/L, TBIL: 16.6 μmol/L, PT: 13.4 s, massive ascites), and the cirrhosis was classified as Child-Pugh 9 and ALBI grade 3. We had to interrupt TACE and only gave sorafenib and camrelizumab. Although he received systemic therapy, the tumors still rapidly progressed and we considered the possibility of tumor resistance. Among the tumor markers, AFP was significantly elevated (12,913.0 μg/L). Then, regorafenib (at a dose of 160 mg daily) was given. In September 2020, the patient received conventional TACE (cTACE) once, regorafenib, and camrelizumab. So far, he had received conversion therapy for half a year, and the treatment effect was not satisfactory. He returned to the local hospital to receive regorafenib every day and camrelizumab once every 3 weeks.

**FIGURE 3:**
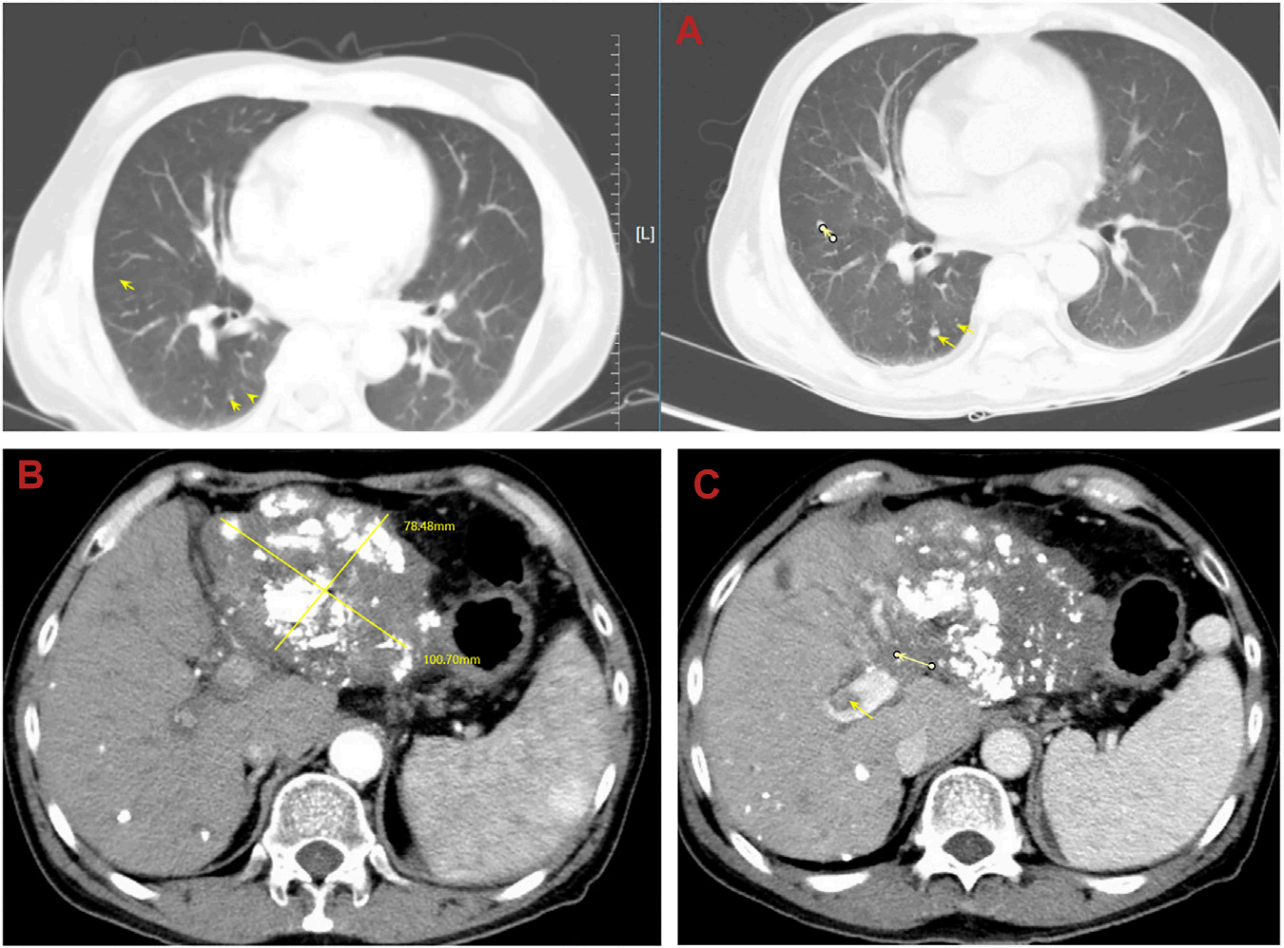
Abdominal computed tomography (CT) shows that multiple small nodules in both lungs were significantly reduced and shrunken and the tumor in the liver was significantly reduced. **(A)** The lung metastases was reduced and shrunken; **(B)** the tumor in the left lobe of the liver shrunk; **(C)** the tumor thrombosis in the left portal vein.

**FIGURE 4:**
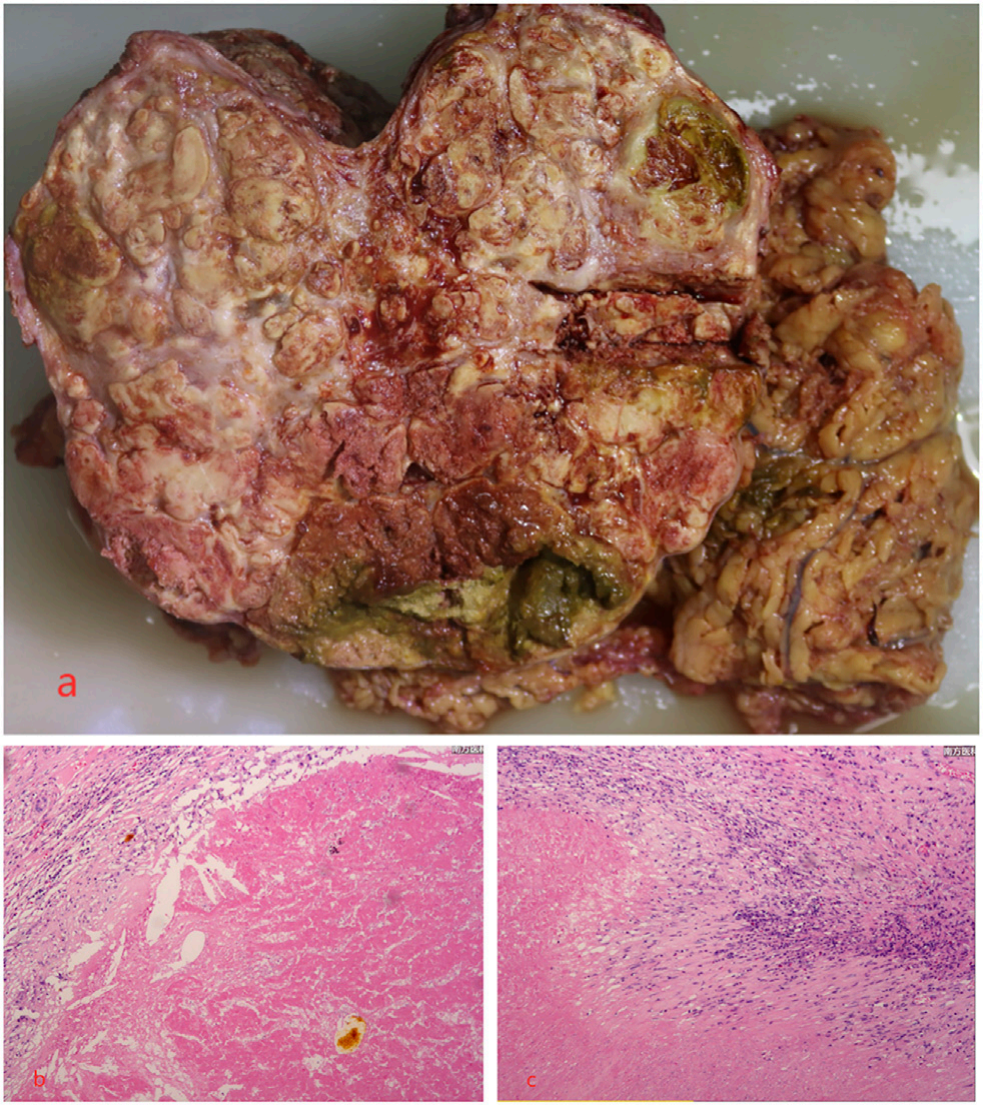
Microscopic examination of the tumor revealed necrosis of the massive HCC with no tumor cells observed on the resection margins, and the portal vein tumor thrombus had necrotic tissue. **(A)** Necrosis of massive HCC; **(B,C)** the pathological examination.

**FIGURE 5:**
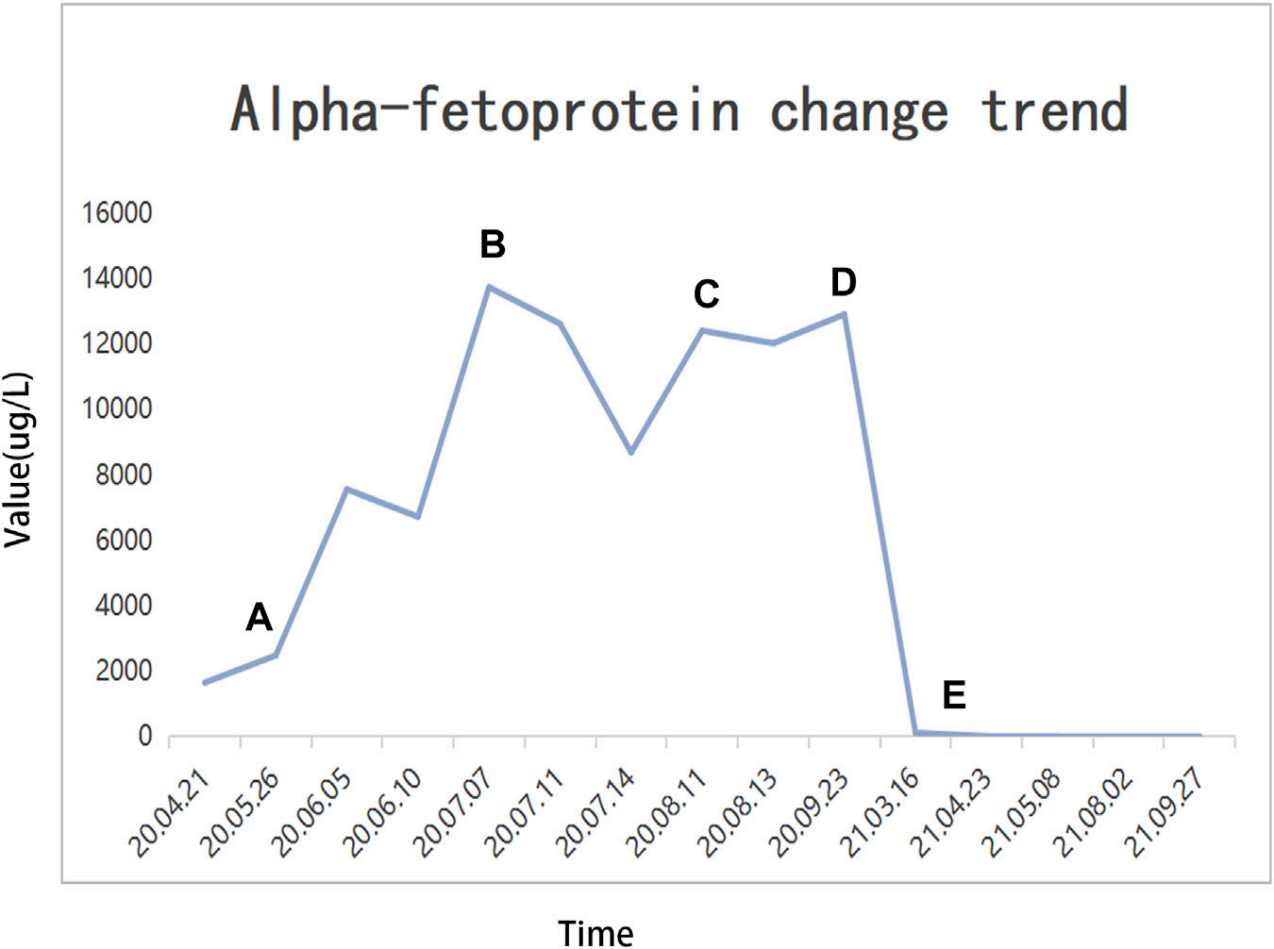
Alpha fetoprotein (AFP) change trend and treatment options. **(A)** TAE + HAIC + sorafenib + camrelizumab; **(B)** dTACE + sorafenib + camrelizumab; **(C)** sorafenib-regorafenib + camrelizumab; **(D)** cTACE + regorafenib + camrelizumab; **(E)** radical surgical resection.

Until a year after his initial diagnosis, the patient found that the tumor and lung metastases had shrunk significantly in the local hospital. Then, he was admitted to our hospital again. On admission, physical examination showed no positive signs. Laboratory data were recorded as follows: ALT: 19 IU/L, AST: 27 IU/L, ALB: 34.2 g/L, TBIL: 8.4 μmol/L, and PT: 11.1 s. AFP was negative (8.1 μg/L). The cirrhosis was classified as Child-Pugh 5 and ALBI grade 2. The enhanced CT revealed there were multiple small nodules in both lungs, which were significantly reduced and shrunken compared to the previous CT. The tumor in the left lobe of the liver was significantly reduced, with local enhancement, and lipiodol deposition was seen. Multiple nodules in the right lobe of the liver had shrunk. And tumor thrombus had formed in the left and right branches of the portal vein (Figure 3). Based on these results, we judged that the patient had partial response (PR) and could be treated with radical surgery, according to the response evaluation in the mRECIST criteria. Then he received left hepatectomy, cholecystectomy, and portal vein incision and embolectomy. The pathological examination showed necrosis of a massive HCC with no tumor cells observed on the resection margins, and without metastasis in lymph nodes. The pathological diagnosis showed that the portal vein tumor thrombus had necrotic tissue (Figure 4). In the patient’s postoperative maintenance therapy, the patient was given lenvatinib 8 mg orally once a day, and camrelizumab 200 mg intravenously once every 3 weeks. There was no clinical evidence of recurrence at 6 months after the resection. The AFP levels remained within the normal limits (Figure 5).

## Discussion

With the development of diagnosis and treatment of HCC, overall survival time has been prolonged. As we all known, surgical resection is the best treatment for liver cancer patients, which can lead to a 60%–70% chance of 5-year survival (Llovet et al., 2003). However, in China, even in the Asia-Pacific region, most patients were found to be at an advanced stage, and more aggressive approaches (systemic therapy, local therapy, targeted therapy, and immunotherapy) were preformed. The prognosis of patients from the Asia-Pacific region is still worse than those from North America or Europe (Cheng et al., 2009). The prognosis for advanced HCC (unresectable or metastatic) remains poor, with a median overall survival of 4.2 months for those without treatment (Cheng et al., 2009). As for HCC with Okuda stage II, the median survival time was 2.7 months (Yeung et al., 2005). Our case reported that the patient with lung metastasis and PVTT successfully underwent radical surgery after conversion therapy. It may provide an alternative treatment method for advanced HCC patients.

For HCC with PVTT, some studies recommend that an aggressive approach may lead to improved survival, and the patients who underwent radical surgery had the best survival, followed by HAIC and cTACE (Takizawa et al., 2007; Lin et al., 2011). When radical surgery cannot be performed, HAIC and cTACE are often used as the first-line treatment, especially in eastern countries (Korean Liver Cancer Study Group, 2014; Kudo et al., 2015; Omata et al., 2017). Liu BJ et al. found that cTACE combined with HAIC (cTACE-HAIC) would achieve longer overall survival (OS) than cTACE alone (median OS 9 vs. 5 months). For HCC patients with PVTT (Vp3-Vp4), the median OS was significantly different between the cTACE-HAIC group (10 months) and cTACE group (4 months) (Liu et al., 2021). A retrospective study that compared cTACE-HAIC and cTACE in the treatment of 83 patients with potentially resectable liver cancer by Yuan YF et al. demonstrated that the surgical conversion rate (48.8% vs 9.5%), mRECIST-objective response rate (ORR) (65.9% vs 16.7%), and progression-free survival (PFS) (HR = 0.38, 95%CI: 0.20–0.70) were better in patients in the cTACE-HAIC group than in the cTACE group (Chen et al., 2021).

When sorafenib, a small molecule that inhibits tumor cell proliferation and tumor angiogenesis, is widely used clinically, the survival time of patients with advanced HCC is prolonged (Wilhelm et al., 2004). A multicenter, phase III, double-blind, placebo-controlled trial of sorafenib conducted by Llovet JM et al. reported that median OS in the sorafenib group was significantly longer than the placebo group (10.7 vs. 7.9 months) (Llovet et al., 2008). A phase III randomized, double-blind, placebo-controlled trial in the Asia-Pacific region found that median OS was 6.5 months in patients treated with sorafenib, compared with 4.2 months for those who received placebo (Cheng et al., 2009). Therefore, sorafenib was recommended to be used with caution in patients classified as Child-Pugh A (Omata et al., 2017). Although sorafenib has improved the survival rate of the patients with advanced HCC, it still cannot disguise the disappointing prognosis. So, regorafenib, a novel multi-kinase inhibitor, came into being, which has more potent inhibitory activities against multiple angiogenic pathways and oncogenic pathways than sorafenib (Wilhelm et al., 2011). Some research studies suggested that sequential therapy with sorafenib and regorafenib can be considered to prolong survival time. In the pivotal RESORCE trial, researchers reported median OS, median PFS, and median time to progression (TTP) of 10.6, 3.1, and 3.2 months, respectively (Bruix et al., 2017). A multicenter retrospective study in Japan described the median OS, PFS, and TTP in patients who received sequential therapy with sorafenib and regorafenib for advanced HCC to be 17.3, 6.9, and 6.9 months, respectively (Ogasawara et al., 2020). Another real-world sequential therapy program with sorafenib and regorafenib for advanced HCC conducted in Korea observed median OS, PFS, and TTP of 10.0, 2.7, and 2.6 months, respectively (Lee et al., 2021). Asia-Pacific guidelines also recommended regorafenib as a second-line treatment (Omata et al., 2017).

Due to treatment advancement, immunotherapy is an emerging area. A major breakthrough included antibodies targeting negative regulators of T cells, which restore the cytotoxic ability of T cells to destroy tumor cells by blocking the programmed death (PD)-1 receptor. Camrelizumab is one of the PD-1 inhibitors, which was made in China. In a multicenter, open-label, parallel-group, randomized, phase II trial, the researchers reported that objective response occurred in 14.7% of 217 patients and the overall survival probability at 6 months was 74.4% (Qin et al., 2020). Moreover, some studies reported that camrelizumab combined with target therapy or chemotherapy can synergistically increase efficacy in advanced HCC (Qing et al., 2021). Wei F et al. demonstrated that the median PFS of the lenvatinib plus camrelizumab group was significantly longer than that of the lenvatinib group (8.0 vs 4.0 months). Additional, lenvatinib plus camrelizumab had the higher ORR (28.57% vs 7.41%) and disease control rate (DCR) (71.43% vs 51.85%) (Wei et al., 2021). Yuan G et al. compared the efficacy and tolerability of camrelizumab and apatinib (C + A) for HCC patients with PVTT. They described that the ORR and DCR were 44.0% and 75.0%, respectively and thought C + A was a promising treatment for HCC with PVTT (Yuan et al., 2020). A multicenter phase Ib/II study about camrelizumab combined with FOLFOX4 for advanced HCC found that the ORR and DCR were 29.4% and 79.4%, respectively. The median duration of response (DOR), PFS, and OS were 6.9, 7.4, and 11.7 months, respectively (Li et al., 2021).

Regarding conversion therapy, there are still some difficult questions to answer, and one is how long the treatment is needed in order to judge the effect. An expert consensus believes that ICIs combined with TKIs have a high surgical conversion rate, and patients with effective treatment generally can be successfully converted after 3-7 treatment cycles (about half a year) (Alliance of Liver Cancer Conversion Therapy et al., 2021; Professional Committee for Prevention and Control of Hepatobiliary and Pancreatic Diseases of Chinese Preventive Medicine Association, 2021). Surgical indications include the following: Child-Pugh grade A or B liver function; adequate residual liver volume after resection, non-cirrhotic patients ≥35% standard liver volume, and cirrhosis patients ≥45% standard liver volume; ICG 15-min tributary rate <20%; the outflow and inflow tract of the liver were intact after the operation; the biliary tract structure was intact after the operation, and the drainage was smooth; the ECOG-PS score was 0–1; and the ASA rating was not higher than 3 (Alliance of Liver Cancer Conversion Therapy et al., 2021; Professional Committee for Prevention and Control of Hepatobiliary and Pancreatic Diseases of Chinese Preventive Medicine Association, 2021). Another question is whether it is still necessary to use a conversion therapy regimen after surgical resection. If it needs to be used, how long will it continue? One expert consensus suggests that ICIs or ICIs combined with TKIs should continue to be given for 6–12 months from 1 month after surgery (Professional Committee for Prevention and Control of Hepatobiliary and Pancreatic Diseases of Chinese Preventive Medicine Association, 2021). Another expert consensus thinks that we can choose the original plan or part of the drug adjuvant treatment in the original plan as appropriate for >6 months. If there is no tumor recurrence and metastasis after two consecutive imaging examinations, and the tumor markers remain normal for 3 months, consider stopping the drug (Alliance of Liver Cancer Conversion Therapy et al., 2021).

Our case reports a patient who was diagnosed with advanced HCC with PVTT and lung metastasis, and successfully implemented conversion therapy. The patient underwent 1 year of conversion therapy, which is far beyond the guideline and previous reports of half a year of conversion therapy. According to previous reports, if patients do not receive effective treatment, the survival time is less than 3 months. However, we used chemotherapy combined with targeted therapy and immunotherapy, and then the patient successfully underwent radical surgery and survived for 6 months without recurrence. For all patients with advanced HCC, this promising result indicates an important step toward a new paradigm of systemic therapy for advanced HCC, and also shows that advanced liver cancer treatment also has the option of radical surgery. Especially, we suggest if the patient is unable to undergo radical surgery and is in a good general condition, the conversion therapy should be as long as possible, which is different from current opinions.

## Conclusion

Now that the treatment of liver cancer has entered the era of targeted therapy combined with immunotherapy, the results obtained from our case show that conversion therapy is promising for tumor control. However, HCC is still a complex disease that requires continuous hard work to improve its prognosis. Further studies are needed to confirm our findings.

## Data Availability

The original contributions presented in the study are included in the article/Supplementary Material, further inquiries can be directed to the corresponding authors.

## References

[B1] Alliance of Liver Cancer Conversion Therapy (2021). Chinese Expert Consensus on Conversion Therapy in Hepatocellular Carcinoma (2021 Edition). Chin. J. Dig. Surg. 20 (6), 600–616.

[B2] Bruix J. Qin S. Merle P. Granito A. Huang Y.-H. Bodoky G. (2017). Regorafenib for Patients with Hepatocellular Carcinoma Who Progressed on Sorafenib Treatment (RESORCE): a Randomised, Double-Blind, Placebo-Controlled, Phase 3 Trial. The Lancet 389 (10064), 56–66. 10.1016/S0140-6736(16)32453-9 27932229

[B3] Chen M. S. Yuan Y. F. Guo R. P. Shi M. Huang J. H. Zhao M. (2021). Application of Hepatic Arterial Infusion Chemotherapy in the Conversion Therapy of Hepatocellular Carcinoma-Eexperience of Sun Yat-Sen University Cancer Center. Chin. J. Front. Med. Sci. 13 (3), 70–76.

[B4] Chen W. Zheng R. Baade P. D. Zhang S. Zeng H. Bray F. (2016). Cancer Statistics in China, 2015. CA: A Cancer J. Clinicians 66 (2), 115–132. 10.3322/caac.21338 26808342

[B5] Cheng A.-L. Kang Y.-K. Chen Z. Tsao C.-J. Qin S. Kim J. S. (2009). Efficacy and Safety of Sorafenib in Patients in the Asia-Pacific Region with Advanced Hepatocellular Carcinoma: a Phase III Randomised, Double-Blind, Placebo-Controlled Trial. Lancet Oncol. 10 (1), 25–34. 10.1016/S1470-2045(08)70285-7 19095497

[B6] He M. Li Q. Zou R. Shen J. Fang W. Tan G. (2019). Sorafenib Plus Hepatic Arterial Infusion of Oxaliplatin, Fluorouracil, and Leucovorin vs Sorafenib Alone for Hepatocellular Carcinoma with Portal Vein Invasion. JAMA Oncol. 5 (7), 953–960. 10.1001/jamaoncol.2019.0250 31070690 PMC6512278

[B7] Korean Liver Cancer Study Group (2014). 2014 Korean Liver Cancer Study Group-National Cancer Center Korea Practice Guideline for the Management of Hepatocellular Carcinoma. Korean J. Radiol. 16 (3), 465–522. 10.3348/kjr.2015.16.3.465 PMC4435981 25995680

[B8] Kudo M. Kitano M. Sakurai T. Nishida N. (2015). General Rules for the Clinical and Pathological Study of Primary Liver Cancer, Nationwide Follow-Up Survey and Clinical Practice Guidelines: The Outstanding Achievements of the Liver Cancer Study Group of Japan. Dig. Dis. 33 (6), 765–770. 10.1159/000439101 26488173

[B9] Lee M. J. Chang S. W. Kim J. H. Lee Y.-S. Cho S. B. Seo Y. S. (2021). Real-world Systemic Sequential Therapy with Sorafenib and Regorafenib for Advanced Hepatocellular Carcinoma: a Multicenter Retrospective Study in Korea. Invest. New Drugs 39 (1), 260–268. 10.1007/s10637-020-00977-4 32749658

[B10] Li H. Qin S. Liu Y. Chen Z. Ren Z. Xiong J. (2021). Camrelizumab Combined with FOLFOX4 Regimen as First-Line Therapy for Advanced Hepatocellular Carcinomas: A Sub-cohort of a Multicenter Phase Ib/II Study. Dddt 15, 1873–1882. 10.2147/DDDT.S304857 33976538 PMC8106453

[B11] Lin D.-X. Zhang Q.-Y. Li X. Ye Q.-W. Lin F. Li L.-L. (2011). An Aggressive Approach Leads to Improved Survival in Hepatocellular Carcinoma Patients with portal Vein Tumor Thrombus. J. Cancer Res. Clin. Oncol. 137 (1), 139–149. 10.1007/s00432-010-0868-x 20340033 PMC3015200

[B12] Liu B.-J. Gao S. Zhu X. Guo J.-H. Kou F.-X. Liu S.-X. (2021). Combination Therapy of Chemoembolization and Hepatic Arterial Infusion Chemotherapy in Hepatocellular Carcinoma with Portal Vein Tumor Thrombosis Compared with Chemoembolization Alone: A Propensity Score-Matched Analysis. Biomed. Res. Int. 2021, 1–13. 10.1155/2021/6670367 PMC8298162 34337041

[B13] Llovet J. M. Burroughs A. Bruix J. (2003). Hepatocellular Carcinoma. The Lancet 362 (9399), 1907–1917. 10.1016/s0140-6736(03)14964-1 14667750

[B14] Llovet J. M. Ricci S. Mazzaferro V. Hilgard P. Gane E. Blanc J.-F. (2008). Sorafenib in Advanced Hepatocellular Carcinoma. N. Engl. J. Med. 359 (4), 378–390. 10.1056/NEJMoa0708857 18650514

[B15] Ogasawara S. Ooka Y. Itokawa N. Inoue M. Okabe S. Seki A. (2020). Sequential Therapy with Sorafenib and Regorafenib for Advanced Hepatocellular Carcinoma: a Multicenter Retrospective Study in Japan. Invest. New Drugs 38 (1), 172–180. 10.1007/s10637-019-00801-8 31172442

[B16] Omata M. Cheng A.-L. Kokudo N. Kudo M. Lee J. M. Jia J. (2017). Asia-Pacific Clinical Practice Guidelines on the Management of Hepatocellular Carcinoma: a 2017 Update. Hepatol. Int. 11 (4), 317–370. 10.1007/s12072-017-9799-9 28620797 PMC5491694

[B17] Professional Committee for Prevention and Control of Hepatobiliary and Pancreatic Diseases of Chinese Preventive Medicine Association (2021). Chinese Expert Consensus on Conversion Therapy of Immune Checkpoint Inhibitors Combined Antiangiogenic Targeted Drugs for Advanced Hepatocellular Carcinoma (2021 Edition). Chin. J. Hepatobiliary Surg. 27 (4), 241–251.

[B18] Qin S. Ren Z. Meng Z. Chen Z. Chai X. Xiong J. (2020). Camrelizumab in Patients with Previously Treated Advanced Hepatocellular Carcinoma: a Multicentre, Open-Label, Parallel-Group, Randomised, Phase 2 Trial. Lancet Oncol. 21 (4), 571–580. 10.1016/S1470-2045(20)30011-5 32112738

[B19] Qing X. Xu W. Zong J. Du X. Peng H. Zhang Y. (2021). Emerging Treatment Modalities for Systemic Therapy in Hepatocellular Carcinoma. Biomark Res. 9 (1), 64. 10.1186/s40364-021-00319-3 34419152 PMC8380325

[B20] Sato Y. Ohnuma H. Nobuoka T. Hirakawa M. Sagawa T. Fujikawa K. (2017). Conversion Therapy for Inoperable Advanced Gastric Cancer Patients by Docetaxel, Cisplatin, and S-1 (DCS) Chemotherapy: a Multi-Institutional Retrospective Study. Gastric Cancer 20 (3), 517–526. 10.1007/s10120-016-0633-1 27553665

[B21] Sung H. Ferlay J. Siegel R. L. Laversanne M. Soerjomataram I. Jemal A. (2021). Global Cancer Statistics 2020: GLOBOCAN Estimates of Incidence and Mortality Worldwide for 36 Cancers in 185 Countries. CA A. Cancer J. Clin. 71 (3), 209–249. 10.3322/caac.21660 33538338

[B22] Takizawa D. Kakizaki S. Sohara N. Sato K. Takagi H. Arai H. (2007). Hepatocellular Carcinoma with portal Vein Tumor Thrombosis: Clinical Characteristics, Prognosis, and Patient Survival Analysis. Dig. Dis. Sci. 52 (11), 3290–3295. 10.1007/s10620-007-9808-2 17394062

[B23] Wei F. Huang Q. He J. Luo L. Zeng Y. (2021). Lenvatinib Plus Camrelizumab versus Lenvatinib Monotherapy as Post-Progression Treatment for Advanced Hepatocellular Carcinoma: A Short-Term Prognostic Study. Cmar 13, 4233–4240. 10.2147/CMAR.S304820 PMC8166816 34079375

[B24] Wilhelm S. M. Carter C. Tang L. Wilkie D. McNabola A. Rong H. (2004). BAY 43-9006 Exhibits Broad Spectrum Oral Antitumor Activity and Targets the RAF/MEK/ERK Pathway and Receptor Tyrosine Kinases Involved in Tumor Progression and Angiogenesis. Cancer Res. 64 (19), 7099–7109. 10.1158/0008-5472.CAN-04-1443 15466206

[B25] Wilhelm S. M. Dumas J. Adnane L. Lynch M. Carter C. A. Schütz G. (2011). Regorafenib (BAY 73-4506): a New Oral Multikinase Inhibitor of Angiogenic, Stromal and Oncogenic Receptor Tyrosine Kinases with Potent Preclinical Antitumor Activity. Int. J. Cancer 129 (1), 245–255. 10.1002/ijc.25864 21170960

[B26] Yeung Y. P. Lo C. M. Liu C. L. Wong B. C. Fan S. T. Wong J. (2005). Natural History of Untreated Nonsurgical Hepatocellular Carcinoma. Am. J. Gastroenterol. 100 (9), 1995–2004. 10.1111/j.1572-0241.2005.00229.x 16128944

[B27] Yuan G. Cheng X. Li Q. Zang M. Huang W. Fan W. (2020). Safety and Efficacy of Camrelizumab Combined with Apatinib for Advanced Hepatocellular Carcinoma with Portal Vein Tumor Thrombus: A Multicenter Retrospective Study. Ott 13, 12683–12693. 10.2147/OTT.S286169 PMC7734076 33328740

